# Leveraging nursery and early childhood education institutions for improving feeding and movement behaviours of infants and young children in China

**DOI:** 10.7189/jogh.14.03011

**Published:** 2024-02-09

**Authors:** Yiwen Huang, Lin Li, Chen Zhao, Peijing Zhong, Xiaotong Wang, Yi Geng, Na Meng, Qiong Wu, Yanfeng Zhang

**Affiliations:** 1 Child Healthcare Center, Children’s Hospital, Capital Institute of Paediatrics, Beijing, China; 2 Beijing KidsHome Children Development Center, Beijing, China; 3 GYMBOREE Educational Information Consultation CO. LTD, Shanghai, China; 4 Department of Integrated Early Childhood Development, Beijing Municipal Key Laboratory of Child Development and Nutriomics, Capital Institute of Paediatrics, Beijing, China

Early childhood represents a critical period for optimal growth and development [1], with the promotion of healthy feeding practices and movement behaviours playing a crucial role in optimising the nutritional status and motor development of infants and young children, ultimately influencing their long-term health outcomes [2]. In 2002, the World Health Organization (WHO) and the United Nations Children’s Fund (UNICEF) jointly issued the Global Strategy on Infant and Young Child Feeding to advocate for best feeding practices. Additionally, in 2019, WHO published guidelines on physical activity, sedentary behaviour, and sleep to enhance movement behaviours for children under five years old [3,4]. Moreover, the Asia-Pacific consensus statement on integrated 24-hour activity guidelines for early childhood provides up-to-date evidence-based recommendations regarding beneficial lifestyle habits for infants and young children [5]. In 2021, China also released physical activity guidelines, including recommendations for children under two years old [6]. However, globally and in China, there is generally suboptimal adherence to recommended feeding practices and movement behaviours. This highlights an urgent need to explore innovative approaches through which caregivers can receive information about feeding practices and movement behaviours. Given the widespread of nursery and early childhood education (ECE) institutions among children under three years old in China, in this paper we aim to discuss the potential utilisation of these institutions for improving feeding practices as well as enhancing appropriate movement behaviours.

## SUBOPTIMAL STATUS OF FEEDING PRACTICES AND MOVEMENT BEHAVIOURS AMONG INFANTS AND YOUNG CHILDREN

Undernutrition is estimated to be associated with 2.7 million child deaths annually, accounting for 45% of all child deaths. Infant and young child feeding is vital in improving child survival and promoting growth and development [7]. However, the feeding practices are generally suboptimal. The exclusive breastfeeding rate of infants younger than six months was 41% worldwide, 37% in low-and middle-income countries, and 34.1% in China [8-10]. Regarding complementary feeding, in 2017, only 28.2%, 50.3%, and 15.9% of children worldwide met minimum dietary diversity, minimum meal frequency, and minimum acceptable diet, respectively [2,11]. Although these indicators were comparatively higher in China at rates of 60.6% for minimum dietary diversity, 72.4% for minimum meal frequency, and 43.4% for minimum acceptable diet, respectively [12], the feeding practices of Chinese children still need further improvement.

Movement behaviours, including tummy time, physical activity, sedentary behaviour and sleep, are crucial for healthy development during early childhood [13,14]. A study in Canada showed that each additional time point of tummy time recommendation adherence was associated with five to 11 days earlier acquisition of independent sitting, crawling, and independent standing milestones [15]. However, compliance with the WHO movement behaviours guidelines remains quite low among children under two years old. Only 30% of children met the WHO’s recommendations for tummy time (30% in the UK, 29.7% in Australia) [15,16], and more than half of children complied with guidelines regarding sedentary behaviour and sleep duration (57.8% and 76.2% in the UK, and 56.9% and 58.7% in Australia) [15,16]. As in China, limited data exists on movement behaviour practices among infants and young children.

## NURSERY AND ECE INSTITUTIONS

Currently, in China, children under three years old are not eligible for kindergarten enrollment and primarily receive care from their families. This requires caregivers to invest significant time and effort and acquire relevant knowledge and operational skills. This situation raises concerns such as unattended childcare for young children and work-family conflicts [17]. Recognising the importance of early childhood development for this age group, in 2019 the Chinese government issued the Guidelines on Promoting the Development of Child Care Services for Infants and Young Children Under 3 Years, emphasising the establishment of a service system that involves local government and community participation, and development of nursery institutions [18]. According to recent data released by the National Health Commission in 2022, 75 700 nursery institutions in China provided services with a capacity for 3 624 000 children and an average provision rate of 2.57 per thousand people [19]. By 2025, the nursery capacity for infants and young children under three years old is projected to increase to 4.5 per thousand of the population [20]. Moreover, a growing number of Chinese parents have attached importance to their children’s early childhood education. Data from ECE associations in 2020 revealed that around 70% of children aged zero to six years in first-tier cities attended ECE institutions, and almost 30% in second-tier provincial capitals, as well as third- and fourth-tier cities [21].

Given the extensive coverage of children, nursery and ECE institutions possess significant potential for enhancing health practices among children under three years old. However, our previous studies have revealed that feeding practices and movement behaviours were suboptimal among children under two years old in ECE institutions. For feeding practices, the prevalence of minimum dietary diversity, minimum meal frequency, and minimum acceptable diet were found to be 59.4%, 60.6%, and 39.2% for children aged six to 23 months old, respectively, which aligns with the national level. However, these rates were much lower for infants aged 6–11 months at 49.1%, 44.9%, and 25.0%, respectively [22]. Regarding movement behaviours, less than half of the children met the WHO’s recommendations on physical activity time (19.2%), physical restraint (45.8%), and screen time (46.4%) [23]. Moreover, 66% and 85.7% of children adhered to WHO’s guidelines on sleep duration and outdoor time during the last 24 hours respectively [23]. Concerning tummy time practice, less than half of infants initiated this activity during their neonatal period (48.2%). Meanwhile, only 27.2% of infants engaged in at least 30 minutes of daily tummy time [24].

## LEVERAGING NURSERY AND ECE INSTITUTIONS

In China, many children under three years old receive care from their families and nurseries or ECE institutions. Therefore, it is essential to strengthen these institutions to provide appropriate services. In 2022, the National Health Commission issued guidelines on infant feeding and nutrition in nursery institutions that guide breastfeeding and complementary feeding [25]. In addition, caregivers should possess knowledge of feeding and movement behaviours. Currently, nursery institutions in China are subject to systematic standardisation encompassing construction, personnel management, and parental training [18,25]. Furthermore, government policies encourage primary health care institutions, maternal and child health hospitals, and traditional Chinese medicine hospitals to establish connections with childcare facilities for guidance provision and staff training [26]. Some ECE institutions incorporated activities into their programs that enhance movement behaviours, such as tummy time and other age-appropriated fitness activities. However, ECE institutions generally lack standardised curricula and are advised to refer to national guidelines for nursery institutions.

Caregivers have access to various health information sources, including family members and friends, health facilities, mass media, and new media platforms. Nursery and ECE institutions can be crucial in educating caregivers through frequent interactions. Traditional offline methods can be effectively employed by encouraging caregivers to participate in face-to-face training sessions. Our previous study also demonstrated the effectiveness of utilising WeChat as a platform for delivering information on feeding and movement behaviours in rural China [27]. Therefore, leveraging the advantages of new media through these institutions should also be considered for caregivers’ health education.

Although guidelines and recommendations are available, and programs have been developed to enhance feeding practices and movement behaviours among young children, behavioural change is challenging due to various implementation issues. Where appropriate, the involvement of non-governmental organisations could offer supplementary support to nursery and ECE institutions owned by governments or operating commercially. Despite the significant potential of these institutions in delivering health information to caregivers, the effectiveness of this delivery channel remains unknown, necessitating further studies.

## CONCLUSIONS

Children under three years old are in crucial developmental stages and receive care both within their families and in nurseries or ECE institutions. Given the extensive coverage of children by these institutions, there is an opportunity to optimise feeding and movement behaviours through curriculum enhancement, staff training, and provision of health information to caregivers. Further studies need to be conducted to evaluate the effectiveness of this delivery channel.
